# Combinatorial strategies based on CRAd-IL24 and CRAd-ING4 virotherapy with anti-angiogenesis treatment for ovarian cancer

**DOI:** 10.1186/s13048-016-0248-5

**Published:** 2016-06-27

**Authors:** Ahmad Mohammad Ashshi, Adel Galal El-Shemi, Igor P. Dmitriev, Elena A. Kashentseva, David T. Curiel

**Affiliations:** Department of Laboratory Medicine, Faculty of Applied Medical Sciences, Umm Al-Qura University, PO Box 7607, Holy Makkah, Saudi Arabia; Department of Pharmacology, Faculty of Medicine, Assiut University, Assiut, Egypt; The Division of Cancer Biology and Biologic Therapeutic Center, Department of Radiation Oncology, School of Medicine, Washington University in Saint Louis, 660 South Euclid Avenue, Campus Box 8224, St. Louis, MO 63110 USA

**Keywords:** Ovarian cancer, Adenovirus, CRAd, Virotherapy, IL-24, ING4

## Abstract

**Background:**

A major hurdle incurrent to the human clinical application of conditionally replicative adenovirus (CRAd)-based virotherapy agents is their limited therapeutic efficacy. In this study we evaluated whether arming our previously reported Ad5/3Δ24 CRAd vector containing a 24-base pair deletion in the E1A conserved region 2, which allows selective replication within Rb-p16-deficient tumor cells, to express therapeutic genes could improve oncolytic virus potency in ovarian cancer cells. We choose to assess the therapeutic benefits achieved by virus-mediated expression of interleukin 24 (IL-24), a cytokine-like protein of the IL-10 family, and the inhibitor of growth 4 (ING4) tumor suppressor protein.

**Results:**

The generated CRAd-IL24 and CRAd-ING4 vectors were tested in ovarian cancer cell lines in vitro to compare their replication, yield, and cytotoxic effects with control CRAd Ad5/3∆24 lacking the therapeutic gene. These studies showed that CRAd-IL24 infection resulted in significantly increased yield of infectious particles, which translated to a marked enhancement of virus-induced cytotoxic effects as compared to CRAd-ING4 and non-armed CRAd. Testing CRAd-IL24 and CRAd-ING4 vectors combined together did not revealed synergistic effects exceeding oncolytic potency of single CRAD-IL24 vector. Both CRAds were also tested along with anti-VEGF monoclonal antibody Avastin and showed no significant augmentation of viral cytolysis by anti-angiogenesis treatment in vitro.

**Conclusions:**

Our studies validated that arming with these key immunomodulatory genes was not deleterious to virus-mediated oncolysis. These findings thus, warrant further preclinical studies of CRAd-IL24 tumoricidal efficacy in murine ovarian cancer models to establish its potential utility for the virotherapy of primary and advanced neoplastic diseases.

## Background

In the past two decades, gene therapy has been developed as a promising approach to combat a variety of diseases. Over this time period, more than 2210 clinical gene therapy trials were conducted, with 64 % addressing cancer [1]. Adenoviral vectors have been used in 22 % of clinical trials, followed by retroviral vectors (18.4 %) and naked/plasmid DNA (17.4 %).

Human adenovirus [2] (Ad) has been used extensively to develop replication-deficient gene delivery vectors and conditionally-replicative Ad (CRAd) agents for cancer treatment. We have previously evaluated several gene therapy strategies including oncolytic CRAd virotherapy for ovarian cancer [3, 4]. This approach takes advantage of the propensity of human Ad to infect and replicate in epithelial cells, the origin of most human cancers, while promoting cell lysis to facilitate release of viral progeny [2]. These features have been exploited by a number of strategies aimed at creating oncolytic CRAd vectors with increased selectivity for cancer cells [5]. The biologic basis of the CRAd’s anti-neoplastic effect is target cell selective replication whereby direct oncolysis achieves specific tumor cell killing. Progeny virions generated in this process may thereby maintain the replicative cycle via lateral infection of adjoining tumor cells. This novel paradigm of amplification has rationalized the rapid translation of CRAd agents to the context of human clinical trials for a variety of neoplastic disease targets.

Of note, we showed that the clinical utility of CRAds derived from Ad serotype 5 (Ad5) for oncolytic treatment of ovarian carcinoma is hampered by inefficient infection of ovarian cancer (OvCa) cells. This is due to the paucity of coxsackievirus group B and Ad receptor (CAR), the primary Ad5 receptor [6–8]. To confer CAR-independent virus tropism we used genetic incorporation of RGD-4C (Cys-Asp-Cys-Arg-Gly-Asp-Cys-Phe-Cys) targeting peptide into Ad5 fiber knob domain [9]. Alternatively, we have endeavored replacement of the knob for its counterpart from Ad serotype 3 [10] that recognizes an alternative receptor, desmoglein 2 [11], a receptor which appears to be more abundantly expressed in ovarian cancer cells [12, 13]. These capsid modifications were employed to alter tropism of CRAd Delta-24 [14], which contains a 24-base pair deletion in the E1A conserved region 2 (CR-2) allowing selective replication within Rb-p16-deficient tumor cells [15]. Of note, this is a defect observed in most ovarian cancer cells [16, 17]. We showed that Delta24-RGD and Ad5/3∆24 CRAd derivatives exhibit superior anti-tumor efficacy in murine models of carcinoma of the ovary [18–20]. On this basis, we have carried out Phase I human clinical trials with both agents whereby their safety has been validated [21, 22]. In the aggregate, these human studies have highlighted the overall safety of CRAd-based interventions.

However, a single modality approach may not be sufficient to eradicate cancer in a patient, because most cancers arise from abnormalities in multiple genetic and signal transduction pathways. To overcome this CRAd shortcoming new oncolytic agents were engineered to contain therapeutic transgenes encoding an apoptosis-inducing and immunomodulatory cytokines. Melanoma differentiation associated gene 7 (MDA-7)/IL-24, a secreted protein of the IL-10 family, functions as a cytokine at normal physiological levels expressed in tissues of the immune system, has shown a great potential as an anti-cancer gene [23]. At supra-physiological levels, MDA-7/IL-24 plays a prominent role in inhibiting tumor growth, invasion, metastasis and angiogenesis resulting in selective cancer cell death without affecting normal cells [24]. The expression of IL-24 mediated by replication-deficient Ad vectors was shown to activate multiple proapoptotic pathways, culminating in decreased ovarian tumor cell survival [25–27]. The inhibitor of growth (ING) family proteins have been defined as candidate tumor suppressors [28]. A novel member of ING family ING4 has potential suppressive effect on different tumors via multiple pathways [29–33]. The use of replication-deficient Ad vectors to express ING4 gene demonstrated improved therapeutic efficacy and growth suppression of lung, pancreatic, and breast carcinoma tumor xenografts [34–36]. Enhanced tumor suppression was shown by nonreplicating bicistronic Ad vector expressing both ING4 and IL-24 genes employed for treatment of human non-small cell lung cancer, breast cancer, and hepatocarcinoma subcutaneous tumor xenografts [37–40]. These studies suggest that arming CRAd vector with tumor suppressors such as ING4 and IL-24 may constitute a novel and effective therapeutic strategy for cancer virotherapy. It was previously shown that Ad5-based CRAd vector engineered to contain the E1A gene under transcriptional control of the promoter region of rodent progression elevated gene-3 and simultaneously express IL-24 gene in place of the deleted E3 region, referred to as cancer terminator virus (CTV), eradicates both primary and distant human breast carcinoma, melanoma, and therapy-resistant prostate cancer cells xenotransplanted in athymic nude mice [41–45]. Our recent studies using novel fiber-chimeric Ad.5/3-CTV CRAd demonstrated improved CAR-independent infection efficiency in low CAR human prostate cancer cells as compared to Ad.5-CTV in vitro while resulting in potent suppression of tumor xenograft growth in a nude mouse model [46, 47] and in a spontaneously induced prostate cancer in Hi-myc transgenic mice [48].

Based on these encouraging data we constructed Ad5/3∆24 CRAd derivatives armed with IL-24 or ING4 gene and tested their oncolytic potency in human ovarian cancer cell lines and immortalized normal ovarian surface epithelial cells. Herein we present evaluation of the generated CRAd-ING4 and CRAd-IL24 vectors either alone, or together, as compared to control CRAd Ad5/3∆24 lacking the therapeutic gene. Armed and control CRAds were also tested along with anti-VEGF monoclonal antibody Avastin (Bevacizumab) to see whether their tumoricidal effects could be affected by anti-angiogenesis treatment in vitro. Overall, these studies revealed that oncolytic potency of CRAd-IL24 is significantly increased as compared to control CRAd and CRAd-ING4 thus, providing a strong rationale for further preclinical testing of CRAd-IL24 therapeutic utility against carcinoma of the ovary.

## Methods

### Cells

The 911 human embryonic retinoblasts derived by transformation with a plasmid containing 79–5789 bp of the Ad5 genome [49] were obtained through Crucell Holland B.V. (Leiden, The Netherlands). The human lung carcinoma cell line A549, ovarian adenocarcinoma cell line OVCAR3 and OV-4 were obtained from American Cell Type Culture Collection (ATCC, Manassas, Virginia USA). The human ovarian carcinoma cell line SKOV3.ip1 was obtained from Janet Price (M. D. Anderson Cancer Center, Houston, Tex.). The normal ovarian surface epithelial cells IOSE-120 (Passage 8) and IOSE-523 (Passage 8), which were obtained from healthy women and immortalized with SV40 T/t were received from Canadian OvCaRe Cell Bank (Vancouver, B.C., Canada). The IOSE-120 and IOSE-523 cells were maintain in a combination of 199 (Sigma M5017) and MCDB105 (Sigma M6395) medium (1:1) supplemented with 5 % FBS and 50 μg/ml gentamicin. All cell lines were grown at 37 °C in medium recommended by the suppliers in a humidified atmosphere of 5 % CO_2_.

### Construction of CRAd vectors

The construction of Ad5/3Δ24 CRAd, which contains deletion of 24 nucleotides (bp 923 – 946) corresponding to the amino acid sequence 122LTCHEAGF129 of the E1A protein necessary for Rb protein binding [14] and has the Ad serotype 3 knob domain incorporated into the Ad5 fiber, was described previously [19, 22]. The genome of CRAd-IL24 armed with human IL-24 was generated as follows. First, the expression cassette containing IL-24 gene under transcriptional control of human cytomegalovirus (CMV) immediate-early promoter/enhancer and followed by synthetic polyadenylation signal, which was constructed as we described previously [48] was cloned into pE3B shuttle plasmid [50] between *BamH*I and *Sal*I restriction sites. The constructed pE3BzCMV-IL24 plasmid DNA was linearized and used for homologous recombination with plasmid carrying CRAd Ad5/3Δ24 genome [51] in *E*. coli BJ5183 cells as described elsewhere [50]. The resultant plasmid carrying the recombinant Ad5/3Δ24cmvIL24 genome containing the CMV promoter-driven IL-24 gene in place of the deleted E3B region was selected using zeocin, cut with *Pac*I to release CRAd-IL24 genome to transfect 911B cells. To construct the genome of CRAd-ING4 armed with *homo sapiens* ING4 we used shuttle plasmid pE3BzCMV-ING4 containing CMV promoter driving the expression of ING4 mRNA transcript isoform 9 (Accession No. NM_001127582), which was synthesized by GenScript USA Inc. (ORF sequence 750 bp, Clone ID: OHu26376C). The pE3BzCMV-ING4 plasmid DNA was linearized and used for homologous recombination with plasmid carrying CRAd Ad5/3Δ24 genome to generate the recombinant Ad5/3Δ24cmvING4 genome as described above. To construct the genome of non-armed CRAd control we used plasmid pCMV-GLuc2 (New England BioLabs Inc., Ipswich, MA USA) that encodes the secreted luciferase (Gluc) from the copepod *Gaussia princeps* to excise the Gluc reporter gene and clone it under CMV promoper in pE3B shuttle plasmid. The constructed pE3BzCMV-Gluc plasmid was linearized and used for homologous recombination with plasmid carrying CRAd Ad5/3Δ24 genome to generate the recombinant Ad5/3Δ24cmvGluc genome as described above. The generated Ad5/3Δ24cmvIL24, Ad5/3Δ24cmvING4, and Ad5/3Δ24cmvGluc plasmids were digested with *Pac*I to release viral genomes to transfect 911B cells and rescue CRAd-IL24, CRAd-ING4, and non-armed control CRAd, respectively.

The newly rescued CRAd vectors were propagated on A549 cells, purified by centrifugation on CsCl gradients according to standard protocol, and dialyzed against phosphate-buffered saline (PBS) (8 mM Na_2_HPO_4_, 2 mM KH_2_PO_4_ [pH 7.4], 137 mM NaCl, 2.7 mM KCl] containing 10 % glycerol. The titers of physical viral particles (vp) were determined by the methods of Maizel et al. [52]. The titers of infectious viral particles were determined by plaque assay using 293 cells as described by Mittereder et al. [53]. The ratios of viral particles to plaque-forming units determined for CRAd-IL24, CRAd-ING4, and CRAd control were 20, 30, and 25 respectively.

### Western blot and ELISA

Samples of SKOV3ip.1 cells infected with CRAd-ING4 at the MOIs of 100, 33, and 11 vp/cell or uninfected control cells were boiled in Laemmli loading buffer and were loaded on a 4–20 % gradient SDS-PAGE gel (Pierce, Rockford, IL). Electrophoretically resolved proteins were transferred to a polyvinylidene fluoride membrane and analyzed for the presence of ING4 polypeptides using polyclonal rabbit antibody raised against ING4 internal region (Assay Biotechnology Company, Inc) diluted 1:1000 for overnight incubation at 4 °C. Bound rabbit antibodies were detected with a secondary goat anti-rabbit or goat anti-mouse antibody conjugated with alkaline phosphatase (Sigma, St. Louis, MO) and developed with alkaline phosphatase substrate kit (Bio-Rad Laboratories, Hercules, CA). The expression of IL-24 was confirmed using OmniKine™ Human IL-24 ELISA kit as recommended by the manufacturer (Assay Biotechnology Company, Inc.) to detect and quantify IL-24 concentrations in culture medium collected from cells infected with CRAd-IL24 at the MOIs of 10 and 1 vp/cell or uninfected control cells.

### CRAd genome quantification

Monolayers of SKOV3ip.1, SKOV3luc, OV-4, and OVCAR3 OvCa cells plated in 6-well tissue culture plates at 5 × 10^5^ cells/well were infected with each CRAd vector at the MOI of 10 vp/cell. Total DNA was purified from the cells harvested from each well 3 days postinfection using QIAamp DNA Mini Kit (QIAGEN, Valencia, CA) as recommended by the manufacturer. The levels of viral genome content were determined in triplicate DNA samples extracted from each cell monolayer by real-time PCR analysis using Light Cycler 480 System (Roche Diagnostics, Indianapolis, IN) with TaqMan primers and probe designed for Ad hexon gene. Resultant viral genome copy number was normalized by amount of cellular DNA, which was determined in the same sample with primers and probe specific for human β-actin (housekeeping gene) using duplexing TaqMan PCR settings.

### CRAd progeny amplification assay

Monolayers of SKOV3ip.1, SKOV3luc, OV-4, and OVCAR3 OvCa cells plated in 6-well tissue culture plates at 5 × 10^5^ cells/well were infected with each CRAd vector at the MOI of 10 vp/cell and incubated for 6 days to obtain complete cytopathic effect (CPE). The monolayers of A549 cells were infected with serial dilutions of lysates of the indicated OvCa cells, which were infected with CRAd-ING4, CRAd-IL24, or control CRAd to determine a cytotoxic endpoint effect (50 % CPE or tissue culture infective dose TCID_50_) 6 days postinfection. The titers of infectious viral progeny produced in each cell line were determined using the Viral ToxGlo assay (Promega Corporation, Madison, WI) to measure cellular ATP level as recommended by the manufacturer.

### Analysis of oncolytic CRAd effects

Monolayers of OvCa cells or immortalized ovarian surface epithelial cells grown in 96-well plates (3 × 10^3^ to 5 × 10^3^ cells/well) were infected in triplicates with CRAd-ING4, CRAd-IL24, or control CRAd at the MOIs ranging from 0.014 to 100 vp/cell. The decrease of cell viability due to the virus-induced cell killing was measured 6 days post-infection using the Cell Proliferation Assay (Promega Corporation, Madison, WI) as recommended by the manufacturer. Assay was performed by adding 10 μL CellTiter 96 AQ_ueous_ One Solution Reagent directly to culture wells containing red phenol red free media supplemented with 2 % FBS, incubating for 2 h and then recording the absorbance at 490 nm with a plate reader (Synergy HT, Bio-Tek Instruments, Winooski, VT). The data are presented as the percentages of viable cells in monolayers infected with each viral dose that were determined with respect to the uninfected control set as 100 %.

To assess the CPE induced by virus propagation cells grown in a 24-well plate (3 × 10^5^ to 5 × 10^5^ cells/well) were infected in triplicates with each CRAd vector at the MOIs ranging from 0.014 to 100 vp/cell. Plates were incubated for 6 days at 37 °C and the cell monolayer integrity was assessed by staining attached cells with crystal violet and then scanning wells using Synergy HT plate reader (Bio-Tek Instruments, Winooski, VT) set at 565 nm. The absorbance values detected in monolayers infected with each viral dose were used to calculate the percentage of cell density in infected cell monolayers with respect to the uninfected control.

To monitor cytotoxic effects induced by each CRAd alone or together the cell monolayers grown in 96-well plates (3 × 10^3^ to 5 × 10^3^ cells/well) were infected with each CRAd alone or two CRAd vectors together at MOI of 1 vp/cell. The infected and uninfected cells were subjected to CellTox™ Green Cytotoxicity assay as recommended by the manufacturer (Promega Corporation, Madison, WI) by adding DNA-binding cyanine dye on day 3 and monitoring the increase in fluorescent signal intensity proportional to cytotoxicity till day 5 post-infection to detect the level of CRAd-mediated cell killing using the Synergy-HT plate reader (Bio-Tek Instruments, Winooski, VT) with 485 nm excitation and 520 nm emission wavelength filters.

### Analysis of cytotoxic CRAd effects combined with Avastin

The monolayers SKOV3ip.1, OV-4, and OVCAR3 OvCa cells were infected with each CRAd alone or two CRAd vectors together at MOI of 10 vp/cell. Avastin obtained through the Alvin J. Siteman Cancer Center pharmacy at Barnes-Jewish Hospital (Washington University School of Medicine, St. Louis) was used to supplement infection and mock infection medium at the concentrations ranging from 0 to 500 μg/ml. The infected and uninfected cells were subjected to CellTox™ assay (Promega Corporation, Madison, WI) by adding DNA-binding cyanine dye on day 3 and monitoring the increase in fluorescent signal intensity till day 5 post-infection to detect cell killing effects. The cells in 96-well plates were red using the Synergy-HT plate reader (Bio-Tek Instruments, Winooski, VT) with 485 nm excitation and 520 nm emission filters.

The Cell Proliferation Assay (Promega Corporation, Madison, WI) was carried out 6 days post-infection by adding 10 μL CellTiter 96 AQ_ueous_ One Solution Reagent directly to culture wells incubating for 1–2 h and then recording the absorbance at 490 nm with a plate reader (Synergy HT, Bio-Tek Instruments, Winooski, VT) to detect cells that stayed alive after exposure to CRAd and/or Avastin. The plates were red using the Synergy-HT plate reader set at 490 nm. The mean values of optical density (OD) detected for each Avastin concentration are presented after subtracting background signal detected in monolayers that were not treated with virus or Avastin.

### Statistical analysis

All data are presented as the mean ± SD. The Student’s two-tailed *t*-test was used to determine statistical significance at the 95 % confidence level, with *p* ≤ 0.05 being considered significantly different.

## Results

### Construction and molecular validation of Ad vectors

**Fig. 1:**
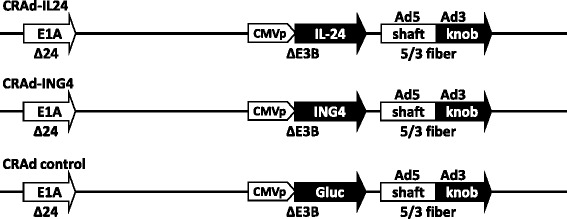
Graphical representation of Ad vectors used in the study. The generated CRAd vectors have a deletion of 24 nucleotides (*Δ24*) in the early E1A gene (*E1A*) to allow selective replication in tumor cells with an pRb mutation. All three vectors encode a chimeric fiber protein (*5/3 fiber*) containing tail and shaft regions of Ad5 fiber fused with knob domain of Ad3, which is known to improve infection efficiency of OvCa cells. CRAd-IL24 (Ad5/3Δ24cmvIL24) contains IL-24 gene incorporated in place of the deleted E3B region (*ΔE3B*) under transcriptional control of the human CMV promoter (*CMVp*). CRAd-ING4 (Ad5/3Δ24cmvING4) is armed with CMVp-driven Inhibitor of Growth 4 (*ING4*) gene. Non-armed CRAd control was constructed to express reporter gene encoding the secreted *Gaussia* Luciferase (*Gluc*)

**Fig. 2:**
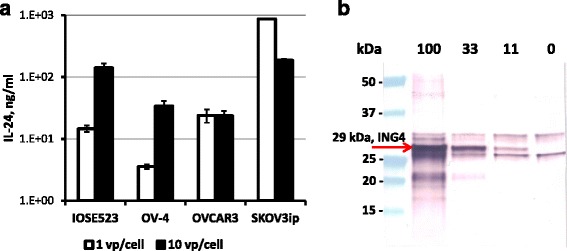
Validation of CRAd-mediated expression of IL-24 and ING4 genes. **a**) The concentrations of IL-24 protein following infection of the indicated OvCa cell lines and normal IOSE523 cells with CRAd-IL24 at the MOIs of 1 and 10 vp/cell were determined in cell culture supernatants 3 days postinfection using commercial ELISA kit with IL-24 concentration standards. Each bar represents the cumulative mean ± SD (*p ≤ 0.05). **b)** The relative levels of ING4 gene expression were determined following infection of SKOV3ip.1 cells with CRAd-ING4 at the MOIs of 100, 33, and 11 vp/cell. The ING4 protein band of 29 kDa was detected in cell lysates 3 days postinfection with 100, 33, and 11 vp/cell, but not in mock-infected cells (0 vp/cell) using Western blot with rabbit polyclonal Ab raised against ING4 internal region

In the current study we designed and constructed three CRAds that are illustrated in Fig. 1. We used the backbone of previously described CRAd Ad5/3Δ24 [19] to incorporate the CMV promoter-driven expression cassette containing either IL-24 or ING4 therapeutic gene within the early E3 region in place of the deleted E3B genes to generate CRAd-IL24 or CRAd-IN4, respectively. The control CRAd was constructed to express the Gluc reporter gene encoding naturally secreted luciferase derived from the copepod, *Gaussia princeps*. The generated CRAd-IL24 and CRAd-IN4 were tested along with control CRAd vector to validate hypothesis that arming Ad5/3Δ24 with IL-24 or ING4 therapeutic payload may improve oncolytic CRAd potency following infection ovarian cancer cells in vitro and in vivo. To this end, the expression of IL-24 and ING4 genes was validated in several ovarian cancer cell lines infected with CRAd-IL24 or CRAd-IN4 vectors. Fig. 2a shows that the presence of IL-24 was confirmed by ELISA at concentrations ranging from 10 to 1000 ng/ml of culture medium collected from ovarian cancer cells OV-4, OVCAR3, SKOV3ip.1, and normal IOSE523 cells, which were infected with CRAd-IL24, but not in mock-uninfected cells (data not shown). Western blot analysis of ING4 expression in SKOV3ip.1 cells infected with CRAd-ING4 at varying MOIs demonstrated a vector dose-dependent increase of 29 kDa protein band intensity, which corresponds to ING4 polypeptide molecular mass as compared to uninfected cells (Fig. 2b). To determine whether arming with IL-24 or ING4 therapeutic genes leads to improved oncolytic CRAd potency we tested cell viability and cytotoxicity following infection of normal and ovarian cancer (OvCa) cells.

### Evaluation of CRAd replication efficacy in vitro

**Fig. 3:**
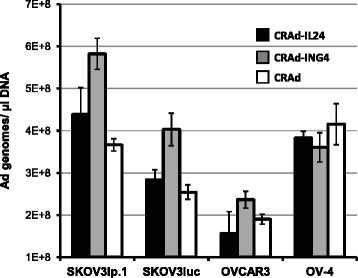
Amplification of CRAd genomes in OvCa cells. The relative amplification of CRAd genome following OvCa cell infection was determined 3 days postinfection using TaqMan qPCR primer/probe to detect viral genome copy number in samples of total DNA isolated from the indicated OvCa cell lines infected with CRAd-IL24, CRAd-ING4, or control CRAd at the MOI of 10 vp/cell. Each bar represents the cumulative mean Ad genome copy number/ng β-actin DNA ± SD (*p ≤ 0.05)

**Fig. 4:**
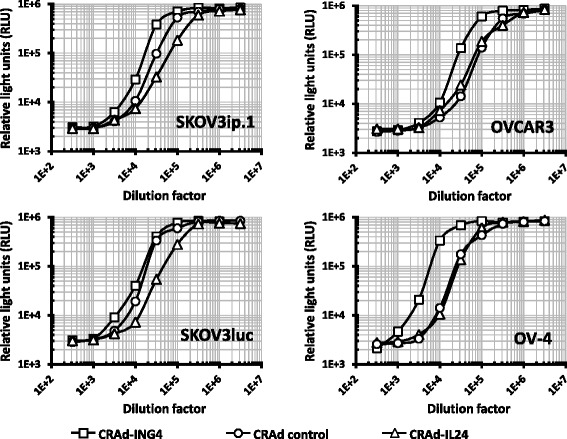
CRAd progeny amplification in OvCa cells. The titers of infectious viral progeny produced in OvCa cell lines were determined using the Viral ToxGlo assay (Promega) to measure cellular ATP level, as surrogate of cell viability. Monolayers of A549 cells were infected with serial dilutions of lysates of the indicated OvCa cells, which were infected with CRAd-ING4, CRAd-IL24, or control CRAd to determine a cytotoxic endpoint effect (50 % CPE or tissue culture infective dose TCID_50_) 6 days postinfection. Each data point represents the cumulative mean ± SD (error bars are smaller than the symbols, *p ≤* 0.05)

To analyze CRAd replication we determined the levels of genome and viral progeny amplification following infection OvCa cells. As illustrated in Fig. 3, CRAd-ING4 vector demonstrated the highest levels of genomic DNA amplification, which was detected in SKOV3ip.1, SKOV3luc and OVCAR3 cells 3 days postinfection using TaqMan qPCR while CRAd-IL24 vector did not show significant differences as compared to control CRAd. To assess the infectious viral progeny produced by CRAd-ING4, CRAd-IL24, or control CRAd following OvCa cells infection the serial dilutions of their lysates were used to inoculate monolayers of A549 cells to determine a cytotoxic endpoint effect (50 % CPE or tissue culture infective dose TCID50) 6 days postinfection. To this end, we employed Viral ToxGlo assay (Promega) to measure luminescent signal intensity dependent on cellular ATP levels, as surrogate of infected cell viability, to detect the decrease in A549 cell viability associated with increased number of infectious CRAd progeny present in serial dilutions of infected OvCa cell lysates. As can be seen in Fig. 4, somewhat similar CRAd-IL24 and control CRAd progeny titers were detected in OVCAR3 and OV-4 cells while CRAd-IL24 infection of SKOV3ip.1 and SKOV3luc cells resulted in significantly increased titers of infectious progeny as compared to control CRAd. Despite the highest viral genomic DNA amplification demonstrated by CRAd-ING4, it showed the lowest infectious progeny titers in all OvCa cells tested. The lack of correlation between viral genome amplification and infectious progeny titers observed following CRAd-ING4 infection suggested that ING4 expression may interfere with infectious viral particles assembly within nucleus of infected cells. In contrast to CRAd-ING4, it seems that amplification of CRAd-IL24 infectious particles was not affected by IL24 gene expression while resulting in infectious progeny titers similar or markedly increased as compared to control CRAd.

### Evaluation of oncolytic effects of CRAd vectors in vitro

**Fig. 5:**
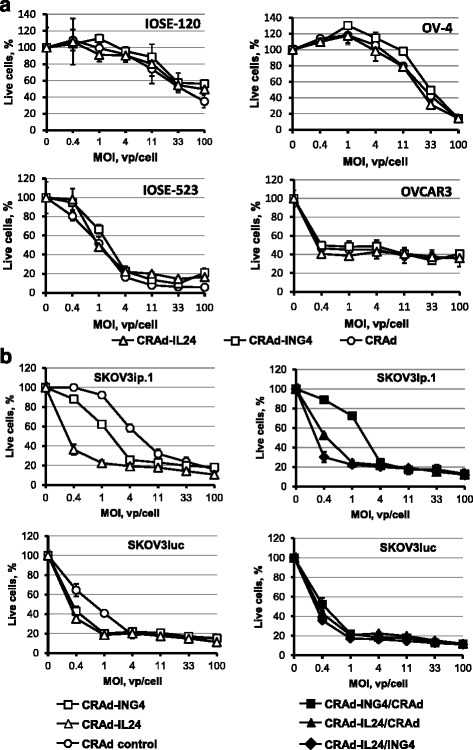
Analysis of cytotoxic CRAd effects in normal and OvCa cells. **a)** The monolayers of normal ovarian surface epithelial IOSE-120 and IOSE-523 cells or OV-4 and OVCAR3 OvCa cells were infected with each CRAd vector at the indicated MOIs (vp/cell). **b)** The monolayers SKOV3ip.1 and SKOV3luc OvCa cells were infected with each CRAd alone (left panel) or two CRAd vectors together (right panel) at the indicated MOIs (vp/cell). The infected and uninfected cells were stained using MTS-based Cell Proliferation Assay (Promega) 6 days postinfection to detect cells that survived CRAd CPE. Live cells percentages are calculated with respect to mock-infected cell monolayers.. Each data point represents the cumulative mean ± SD (some error bars are smaller than the symbols, *p ≤* 0.05)

To detect the effects of IL-24 or ING4 gene expression on CRAd potency we infected monolayers of normal ovarian surface epithelial cells IOSE-120 and IOSE-523 or OvCa cells at MOIs ranging from 0.4 to 100 vp/cell and tested cell viability 6 days postinfection. As can be seen in Fig. 5a, we did not observed any significant differences in proliferation of cells infected with armed CRAds as compared to control CRAd. Both IOSE-523 and OVCAR3 cells were very susceptible to CRAd infection resulting in 50 % live cells at the MOI of 1 and 0.4, respectively as compared to uninfected cells. IOSE-120 and OV-4 were relatively resistant to CRAd infection while showing 50 % cell viability at MOI of 33. Infection of SKOV3ip.1 and SKOV3luc cells revealed significant differences in oncolytic CRAd potency. Both armed CRAds significantly decreased cell viability at MOIs 0.4 and 1 as compared to control CRAd in SKOV3luc cells. Infection of SKOV3ip.1 cells revealed that cytotoxic CRAd-ING4 effects were markedly improved as compared to control CRAd. On the other hand, while CRAd-IL24 vector demonstrated oncolytic potency significantly superior to CRAd-ING4 vector. To see if combining armed CRAds together may result in improved cytotoxicity we tested viability of cells infected with both CRAd-IL24 and CRAd-ING4 vectors as compared to control CRAd along with either CRAd-IL24 or CRAd-ING4. As illustrated in Fig. 5b (right panel), the cytotoxic effects induced in SKOV3ip.1 cells by of CRAd-IL24 and CRAd-ING4 together were somewhat superior to CRAd-IL24 combined with control CRAd and markedly improved with respect to CRAd-ING4 combined with control CRAd. However, the effects observed while using CRAd-IL24 and CRAd-ING4 vector together were not substantially different from those seen with CRAd-IL24 alone at the same MOI (Fig. 5b, left panel). We also could not detect substantial differences between any CRAd combinations used to infect SKOV3luc cells (Fig. 5b, right panel). Despite somewhat dramatic cytotoxicity increase was observed in SKOV3ip.1 cells when CRAd-ING4 was combined with CRAd-IL24 vector as compared to control CRAd these data suggested that employing both armed CRAds together may benefit primarily CRAd-ING4 vector rather than CRAd-IL24 vector.

**Fig. 6:**
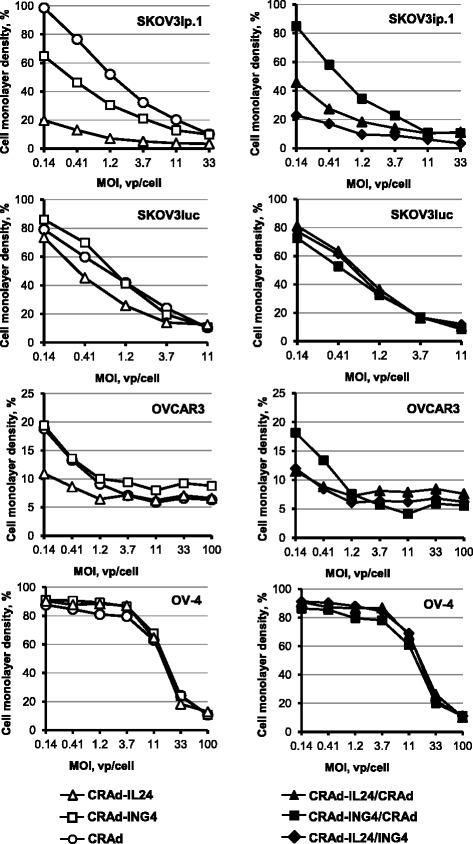
Evaluation of oncolytic effects of CRAd-IL24 or CRAd-ING4 alone and combined together. Monolayers of SKOV3ip.1, SKOV3luc, OVCAR3, and OV-4 cells were infected with each CRAd alone (left panel) or two CRAd vectors together (right panel) at the indicated MOIs. Oncolytic effects of CRAd vectors were assessed by determining cell monolayer integrity 6 days postinfection. The integrity of cell monolayers was determined by staining adherent cells with crystal violet. The stained monolayers were scanned using a plate reader set at 565 nm to calculate the percentage cell density in monolayers infected with each viral dose that were determined with respect to the uninfected control. Each data point represents the cumulative mean ± SD (error bars are smaller than the symbols, *p ≤* 0.05)

Oncolytic CRAd effects were further assessed in OvCa cells after staining adherent cells with crystal violet 6 days postinfection and determining the densities of cell monolayers infected at various MOIs. The values of cell density presented in Fig. 6a show markedly enhanced cytopathic effects of CRAd-IL24 vector as compared to both CRAd-ING4 and CRAd control in SKOV3ip.1 and SKOV3luc cells. While cytopathic effects of CRAd-ING4 vector were significantly increased in SKOV3ip.1 cells we did not detect a marked improvement of cytotoxicity in SKOV3luc cells as compared to control CRAd vector. While combining CRAd-IL24 and CRAd-ING4 vectors produced cytopathic effects somewhat similar to CRAd-IL24 alone in SKOV3ip.1 cells it resulted in a marked loss of cytotoxicity in SKOV3luc cells as compared to CRAd-IL24 alone (Fig. 6a). We observed that cytopathic effects of CRAd-IL24 vector were somewhat increased at lower MOIs in OVCAR3 cells as compared to CRAd-ING4 and control CRAd vector however, no beneficial effects of CRAd arming with IL-24 and ING4 genes were revealed in OV4 cells (Fig. 6b).

**Fig. 7:**
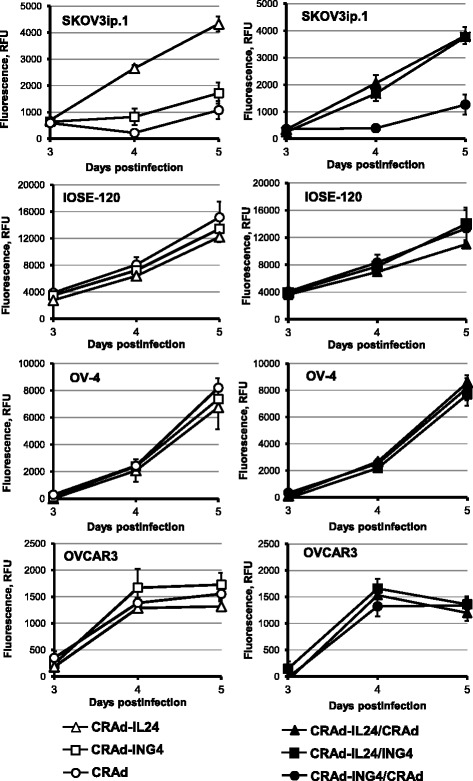
Monitoring cytotoxic effects induced by CRAd-IL24 or CRAd-ING4 alone and together. The monolayers of SKOV3ip.1, OvCa OV-4, or OVCAR3 ovarian cancer cells and normal ovarian surface epithelial IOSE-120 cells were infected with each CRAd alone (left panel) or two CRAd vectors together (right panel) at MOI of 1 vp/cell. The infected and uninfected cells were subjected to CellTox™ Green Cytotoxicity assay (Promega) by adding DNA-binding cyanine dye on day 3 and monitoring the increase in fluorescent signal intensity proportional to cytotoxicity till day 5 postinfection to detect the level of CRAd-mediated cell killing using the Synergy-HT plate reader with 485 nm excitation and 520 nm emission wavelength filters. Each data point represents the cumulative mean ± SD (some error bars are smaller than the symbols, *p ≤* 0.05)

To monitor CRAd-mediated cell killing effects we employed CellTox™ Green Cytotoxicity assay (Promega). The monolayers OvCa cells and normal IOSE-120 cells were infected with each CRAd alone or two CRAd vectors together at MOI of 1 vp/cell. The changes in citotoxicity were detected by adding DNA-binding cyanine dye and measuring the levels of fluorescent signal intensity in infected and uninfected cells, which is proportional to number of killed cells on day 3, 4 and 5 postinfection (Fig. 7). We observed that infection of SKOV3ip.1 cells with armed CRAds resulted in markedly improved cell killing as compared to control CRAd while no significant differences were detected between armed and control CRAd vectors in IOSE-120, OV-4, and OVCAR3 cells. As can be seen in Fig. 7a, the use of CRAd-IL24 alone or in combination with either CRAd-ING4 or control CRAd resulted in up to 4-fold increased levels of cytotoxicity as compared to control CRAd alone or together with CRAd-ING4, respectively. The use of CRAd-ING4 alone showed only a marginal improvement in cell killing as compared to control CRAd.

### Analysis of CRAd oncolysis in the presence of Avastin

**Fig. 8:**
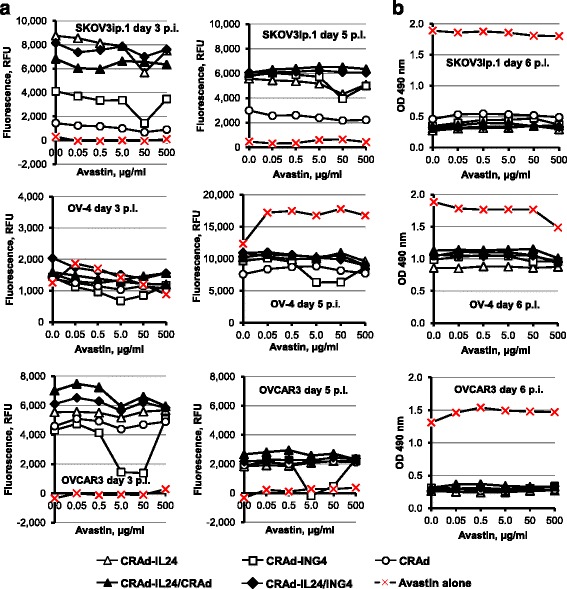
Analysis of cytotoxic CRAd effects combined with Avastin. **a**) The monolayers SKOV3ip.1, OV-4, and OVCAR3 OvCa cells were infected with each CRAd alone or two CRAd vectors together at MOI of 10 vp/cell. Avastin was used to supplement infection and mock infection medium at the indicated concentrations (μg/ml). The infected and uninfected cells were subjected to CellTox™ assay (Promega) by adding DNA-binding cyanine dye on day 3 and monitoring the increase in fluorescent signal intensity, which is proportional to cytotoxicity till day 5 post-infection to detect any cell killing effects. The cells in 96-well plates were red using the Synergy-HT plate reader with 485 nm excitation and 520 nm emission filters. The data represent the mean values of relative fluorescent units (RFU) detected for each Avastin concentration after subtracting background signal detected with cyanine dye added but no virus or Avastin. **b**) The Cell Proliferation Assay (Promega) was carried out 6 days post-infection to detect cells that stayed alive after exposure to CRAd and/or Avastin. The plates were red using the Synergy-HT plate reader set at 490 nm. The mean values of optical density (OD) detected for each Avastin concentration are presented after subtracting background signal detected in monolayers that were not treated with virus or Avastin. Each data point represents the cumulative mean ± SD (error bars are smaller than the symbols, *p ≤* 0.05)

To test tumoricidal effects of CRAd virotherapy combined with anti-VEGF monoclonal antibody (mAb) Avastin (Bevacizumab) we infected SKOV3ip.1, OV-4, and OVCAR3 cells with each CRAd alone or two CRAd vectors together at the MOI of 10 vp/cell at day 0 while supplementing culture medium with Avastin at the concentrations ranging from 0 to 500 μg/ml. (Avastin™; Roche, Switzerland, or Bevacizumab; Genentech BioOncology, South San Francisco, CA, USA). The infected and uninfected cells were subjected to CellTox™ assay (Promega) by adding DNA-binding dye and measuring the fluorescent signal intensity, which is proportional to cytotoxicity on day 3, 4, and 5 post-infection to assess the cell killing effects. Figure 8a (left panel) shows that CRAd-IL24 alone and in combination with either CRAd-ING4 or control CRAd demonstrated markedly increased cell killing efficiency on day 3 postinfection as compared to control CRAd alone. While CRAd-ING4 showed significantly increased cytotoxicity as compared to control CRAd in SKOV3ip.1 cells, it was somewhat decreased in OV-4 and OVCAR3 cells on day 3. When cytotoxic effects were monitored on day 5 postinfection (Fig. 8a, left panel), we did not observe any consistent differences between tested CRAds in OVCAR3 cells while all CRAd combinations showed substantially improved killing of SKOV3ip.1 cells and somewhat increased cytotoxicity (except for CRAd-ING4) in OV-4 cells as compared to control CRAd. Despite the differences that we observed in CRAd-mediated cell killing efficacy, it was not dependent on Avastin concentration suggesting that oncolytic CRAd effects may not benefit from anti-angiogenesis treatment in vitro. These data were corroborated by Cell Proliferation Assay (Promega) carried out 6 days postinfection and showing no dose-dependent effects on a viability of both infected and uninfected cells (Fig. 8b, right column). Overall, these in vitro studies did not reveal any evidence that anti-angiogenesis mAb treatment has direct effect on OvCa cell viability in a wide range of Avastin concentrations tested.

## Discussion

The frequent resistance of aggressive cancers to currently available therapies, such as radiotherapy and chemotherapy, mandates development of targeted, nontoxic and more efficacious treatment modalities. CRAd agents that induce oncolysis by cancer-specific replication are currently being evaluated in clinical trials [54, 55] demonstrating high safety profile but limited clinical efficacy. Further in this regard, arming CRAds with immunomodulatory or proapoptotic genes is being explored as a strategy to enhance their potency.

In this study we generated the derivatives of Ad5/3Δ24 CRAd vector designed to express therapeutic genes that could potentially improve viral oncolysis in ovarian cancer cells. Based on previous encouraging data using IL-24 gene to arm both Ad5-based and Ad5/3-based CTV CRAd [41–48] we choose to test whether our Ad5/3Δ24 vector designed to express IL-24 could provide therapeutic benefit for oncolytic treatment of carcinoma of the ovary. We also thought to assess the utility of CRAd arming with ING4 gene, which was previously shown to provide growth suppression of several tumor types, when being expressed alone [34–36] or together with IL-24 gene [37–40] using replication-incompetent Ad vectors. To this end, we constructed Ad5/3Δ24 CRAd derivatives containing either IL-24 or ING4 gene controlled by constitutive CMV promoter incorporated in place of the early E3B genes deleted within viral chromosome (Fig. 1). Following validation of efficient expression of IL-24 and ING4 genes in OvCa cell lines infected with newly generated CRAds (Fig. 2a, b) we carried out multiple assays to assess the efficiency of virus replication and the extent of induced cytotoxicity.

First, we determined the viral chromosome amplification in OvCa cells infected with CRAd-ING4, CRAd-IL24, control CRAd vector, which revealed that the CRAd-ING4 genome copy numbers were significantly increased as compared to CRAd-IL24 and control CRAd in three out of four OvCa cell types tested (Fig. 3). When the yield of infectious viral progeny produced in these cells was determined based on TCID_50_ assay it appeared that CRAd-ING4 infection resulted in significantly lower titers of infectious virus as compared to control CRAd in all tested OvCa lines. On the other hand, CRAd-IL24 infectious progeny produced in SKOV3ip.1 and SKOV3luc cells was markedly increased with respect to control CRAd (Fig. 4). This discrepancy between viral genome amplification and the resultant amount of infectious viral progeny indicated that overexpression of IL-24 and ING4 genes in infected cells may affect the CRAd genome replication and/or subsequent DNA packaging into fully assembled infectious virions. While previous studies of CTV CRAd derivatives [41–48] expressing IL-24 gene did not report any drawbacks affecting viral progeny amplification and assembly, therapeutic ING4 effects were analyzed in the context of replication-incompetent Ad-based expression [34–36]. It is likely that exogenous ING4 can control cellular pathways interfering with CRAd Δ24 life cycle following cancer cell infection. p29ING4 was reported to interact with p300, a major component of histone acetyl transferase complexes, and negatively regulate the cell growth by decreasing cell population in S-phase, inducing significant G2/M arrest of cell cycle and apoptosis [56, 57]. In contrast to ING4, the E1A proteins facilitate cell cycle progression by binding to the p300 and CBP proteins, which is mediated by CR-1 [58]. E1A mutants lacking CR-2 and unable to bind pRB family members can nevertheless stimulate cellular DNA synthesis to move the infected cells from G1 to S phase allowing efficient viral DNA replication [59, 60]. Therefore, the published data and our own observations strongly suggest that vector-mediated ING4 expression can suppress cancer cell proliferative status thereby, creating an environment unfavorable for CRAd propagation.

To evaluate oncolytic potency of armed CRAd-IL24 and CRAd-ING4 with respect to control CRAd we carried out multiple assays measuring cell viability following infection at a wide range of MOIs. Analysis of cell proliferation and cytotoxicity showed that oncolytic potency of CRAd-IL24 was dramatically improved as compared to non-armed CRAd and superior to CRAd-ING4 in in SKOV3ip.1 cells. While CRAd-IL24 and CRAd-ING4 infection resulted in somewhat similar cytotoxicity increase in SKO3luc cells as compared to control CRAd we did not observe any significant differences between CRAd vectors in OV-4 and IOSE-120/523 cells.

We also tested whether combining CRAd-IL24 and CRAd-ING4 vectors may result in augmentated cytotoxicity in OvCa cells as compared to their separate application. When SKOV3ip.1 cells were infected with CRAd-IL24 mixed with equal amount of either CRAd-ING4 or un-armed CRAd we observed an overall augmentation of cytotoxic effects induced by CRAd-IL24/ING4 mix as compared to CRAd-IL24/CRAd or CRAd-ING4/CRAd mixture. However, the resultant oncolytic effects detected in all tested OvCa cells were not increased above the level induced by the same viral particle number of CRAd-IL24 along. Hence, these experiments did not reveal any significant benefits while combining CRAd/IL-24 and CRAd-ING4 together, thereby, indicating that the predominant pathways that mediate IL-24 and ING4 therapeutic effects are not synergistic in vitro.

Finally, we tested if supplementing infection media with Avastin (Bevacizumab), a recombinant humanised monoclonal antibody developed against soluble VEGF to prevent receptor binding [61], may increase oncolytic potency of CRAd-IL24 and/or CRAd-ING4 in OvCa cells. Interestingly, some recent studies indicate that in addition to inhibition of angiogenesis high doses of Avastin may have direct anti-cancer cell properties in vitro [62]. To address this issue we monitored cytotoxic effects induced by each CRAd alone or two CRAds added together in the presence of increasing concentrations of Avastin (0–300 μg/ml). The cytotoxic effects measured on day 3 and 5 post-infection demonstrated that the CRAd-mediated cell-killing in SKOV3ip.1, OV-4, and OVCAR3 cultures was essentially the same in the absence or presence of Avastin at a wide range of concentrations tested (Fig. 8a). These data were supported by cell viability assay carried on day 6 post-infection, which did not show any significant reduction of cell viability caused by CRAd vectors in the presence of Avastin (Fig. 8b). The treatments of cell monolayers with indicated Avastin concentrations did not reveal significant cytotoxic or proliferative effects except for OV-4 cells at day 5 postinfection, which was not concentration-dependent and could be caused by other factors. Collectively, these data along with previous studies [63] indicate the lack of direct anti-cancer properties in vitro. On the other hand, this assay (Fig. 8a) corroborated our previous observations that CRAd-IL24 oncolytic function is markedly superior to both CRAd-ING4 and control CRAd, thus, validating that observed increase of infectious CRAd-IL24 vector yields (Fig. 4) can translate to enhanced cell-killing efficacy (Figs. 5b, 6, 7a, 8a).

## Conclusions

In the aggregate, our in vitro findings warrant further preclinical studies of CRAd-IL24 to test its efficacy in murine ovarian cancer models to establish its potential utility for the virotherapy treatment of primary and advanced neoplastic diseases.

Whereas oncolytic virotherapy agents were originally designed to accomplish anti-neoplastic effects directly via replicative oncolysis, it has recently become apparent that they can also elicit potent immunogenic tumor cell death. Specifically, virus infection of cancer cells releases damage-associated patterns recognized by receptors expressed on cells of the innate immune system. Activation of these receptors induces pro-inflammatory cytokines provoking Th1-type immune responses [64–66]. The recognition of these potent immunostimulatory effects of virotherapy agents has led to the design of strategies to augment active anti-tumor immunization using armed CRAd agents [67–69]. This recognition now allows us to determine the full utilities that derive from our CRAd-IL24 agent in preclinical studies employing immunocompetent syngenic murine ovarian cancer model [70].

## Abbreviations

Ad, adenovirus; Ad5, adenovirus serotype 5; CAR, coxsackievirus group B and adenovirus receptor; CMV, cytomegalovirus immediate-early promoter; CPE, cytopathic effect; CR-2, conserved region 2; CRAd, conditionally replicative adenovirus; CTV, cancer terminator virus; Gluc, *Gaussia princeps* luciferase; IL-24, interleukin 24; ING4, inhibitor of growth 4 tumor suppressor protein; mAb, monoclonal antibody; MDA-7, melanoma differentiation associated gene 7; MOI, multiplicity of infection; OvCa, ovarian cancer; PBS, phosphate-buffered saline; RGD-4C, Cys-Asp-Cys-Arg-Gly-Asp-Cys-Phe-Cys; VEGF, vascular endothelial growth factor; vp, viral particles
